# Can Intra-Oral Qualitative Sensory Testing Foretell Postoperative Dental Pain? A Preliminary Report

**DOI:** 10.3390/ijerph19138059

**Published:** 2022-06-30

**Authors:** Alona Emodi-Perlman, Deia Altarescu, Pessia Frideman-Rubin, Ilana Eli

**Affiliations:** Department of Oral Rehabilitation, the Maurice and Gabriella School of Dental Medicine, Tel Aviv University, Tel Aviv 6139001, Israel; deia.altarescu@gmail.com (D.A.); pessia80@gmail.com (P.F.-R.); elilana@tauex.tau.ac.il (I.E.)

**Keywords:** postoperative pain, dental, quantitative sensory testing, qualitative sensory testing, QST, QualST, dental restorations

## Abstract

Pain prevention and management is one of the primary goals of dental care. Postoperative dental pain (PDP) following caries removal and performance of a restorative dental treatment is a common clinical phenomenon, often causing significant discomfort to dental patients. In the present study, a psychophysical non-invasive method, qualitative sensory testing (QualST), was used in an attempt to foretell PDP following dental restorative procedures. Forty-two dental patients underwent an intra-oral cold QualST four times: immediately prior to a restorative dental procedure and at a follow-up meeting 1–3 weeks later, on the treated and on the contralateral oral sides. The QualST measures included subjects’ evaluation of the magnitude of pain and cold sensations experienced (on visual analogue scales) and the duration of the cold sensation (in seconds). Additional measures included age, gender, level of dental anxiety, jaw treated, and type of dental restoration performed (Class I or Class V). Subjects’ PDP was assessed through the phone using numeric rating scales 24, 48, and 72 h postoperatively. The highest level of PDP experienced by subjects occurred 24 h postoperatively (ANOVA with repeated measures). Of the study variables, the QualST pain sensation (B = 0.645, *p* < 0.001), duration of the cold sensation (B = 0.042, *p* < 0.05), and an interaction between gender and dental anxiety (B = 0.136, *p* < 0.05) emerged as possible predictors of the highest PDP experienced by subjects (stepwise regression). The results suggest that subjects’ reaction to an intra-oral cold stimulation of the oral mucosa can serve as a potential tool to foretell postoperative dental pain following restorative dental procedures.

## 1. Introduction

Postoperative dental pain (PDP) is a common phenomenon [1]. It has been reported in about 38–75% of adults and children undergoing dental restorative treatment [2,3], with no relation to the type of restoration and/or the restorative material used [3,4,5]. Factors that possibly influence PDP include polymerization shrinkage, depth of restoration, the technique used, gender, age, and dental anxiety [1,2,4,5,6]. PDP may persist for several days and lead to the use of analgesic agents [7]. PDP control is important since an individual’s pain experience is often a determinant of whether patients seek health care in the future. Thus, prediction and treatment of PDP should be an integral part of any professional dental care.

Being a subjective experience, quantifying pain in a standardized way is important to obtain a broader assessment other than self-report with respect to cultural and ethnic diversity [8]. One psychophysical non-invasive method, largely used to assess somatosensory function, is quantitative sensory testing (QST) [9].

Similar to other psychophysical evaluations, QST uses quantitative mechanical, electrical, thermal (cold/heat), or chemical stimuli applied to the skin, muscle, or mucosa [9,10,11,12,13]. In 2006, the somatosensory phenotype of patients with neuropathic pain was characterized by the nationwide multicenter trials of the German Research Network on Neuropathic Pain (DFNS) [14]. In 2011, the international taskforce on somatosensory testing established by Special Interest Group of Oro-facial Pain under the International Association for the Study of Pain (IASP) provided guidelines for comprehensive screening examination procedures in the orofacial region [15].

On the oral mucosa, QST has been proven to be stable and reliable in assessing somatosensory function in healthy volunteers, special pain populations, and patients [9,16,17]. To date, QST has been used to predict the future occurrence or severity of clinical pain and to predict and reflect treatment responses [12,13,14,15,16,17,18]. For example, Nasri-Heir et al., showed a prolonged painful sensation after intraoral cold stimulus in patients suffering from painful post-traumatic trigeminal neuropathy after endodontic treatment [18].

Baad Hansen et al., showed that simple clinical alternatives (referred to as qualitative sensory testing (QualST)), such as the use of a cotton swab to apply touch stimulus, the use of stainless-steel dental spatula to apply cold stimulus, or the use of a dental examination probe to apply pinprick stimulus, show sufficient inter- and intra-examiner reliability and can be used as a screening method [17]. Zaguri et al. [12] used a 5-mm-diameter cotton swab sprayed with ethyl chloride applied to the alveolar mucosa in order to apply a cold stimulus in patients with atypical odontalgia and reported an extended painful after-sensation following the cold stimulus in such patients. The initial hypothesis of the present study was that measures of an intra-oral thermal (cold) QualST (IT-QualST) would be able to foretell PDP levels among dental patients.

Therefore, the aim of the present study was to describe and validate the IT-QualST as a mode to foretell post-procedural pain related to dental restorations.

## 2. Materials and Methods

### 2.1. Population

Patients were recruited from the dental clinics of senior dental students at the School of Dental Medicine, Tel Aviv University. Informed consent was obtained from all participants. This study received the approval of the ethical committee of Tel-Aviv University (approval no. 0000702-1).

Inclusion criteria were age over 18 years and a need to receive an initial Class I or Class V restoration (with a minimal penetration into dentin—“just into dentin”) with no gingival inflammation and/or recession and with an intact contra lateral tooth.

Exclusion criteria were teeth with previous restoration(s), teeth with gingival recessions, restorations of extensive carious lesions, and restorations that required the use of a matrix band. Additionally, patients who reported using analgesic medications prior to or post dental treatment were excluded from the final calculations.

Restorations were performed by senior dental students under close supervision of their clinical instructors. All procedures were strictly standardized, and students were requested to obtain approval after each and every one of the steps involved in the clinical procedure (diagnosis, cavity preparation, cavity cleaning, resin application). The restoration was performed under local anesthesia with the use of composite resin. The restorative procedure included cleaning the cavity with a cavity cleaner (Cholohexidine digluconate 2%, Bisco) and the use of etching and bonding (GC Premio bond 8th generation or 3M single bond, Universal). Restoration in which one (or more) of the procedural steps was not approved by an instructor was not included in the study. All the patients had previous experience with dental care.

The following general information regarding participants was collected: gender, age, jaw treated (maxilla/mandible), sort of restoration (Class V/Class I), use of medications (prior and/or post treatment), and level of dental anxiety. The latter was included in this study to evaluate the possible influence of fear and anxiety on subjects’ perception and report of pain.

In total, 55 patients were approached and requested to participate in the study. Three subjects declined due to fear of pain, two were excluded due to medical conditions that can affect pain perception, and four were excluded due to the use of analgesic drugs prior to treatment (84% response rate). Five subjects were excluded from the study as the performed restoration did not reach dentin and five subjects due to the use of analgesic drugs post treatment (some overlapping between the excluded groups exists). The final study population comprised 42 patients.

### 2.2. Tools

#### 2.2.1. Assessment of Dental Anxiety

Subjects’ level of dental anxiety was assessed through the Dental Anxiety Scale (DAS) [19]. DAS was developed by Corah as a specific measure of dental anxiety and has been widely used since [20,21,22,23,24,25,26,27]. It includes four items, referring to four dentally related situations. Respondents are asked to indicate which option is closest to their likely response to that situation. Items are scored on a scale of 1 (no anxiety whatsoever) to 5 (extreme anxiety) and summed to give an overall anxiety score ranging from 4 to 20 [19]. The questionnaire has been used widely in numerous studies, including in Israel [28,29,30,31].

#### 2.2.2. Intra-Oral Thermal Test (IT-QualST)

As subjects’ ability to process relevant information in a stressful clinical situation might be impaired [32], communication with subjects regarding the aim of this study, nature of the performed tests, and nature of the requested reports was carefully structured. Information was supplied to subjects in the form of a printed page, distributed prior to the first test by the same investigator (DA). The text concerning the possibility of PDP was as follows: “The researcher (DA) will contact you following 24, 48, and 72 h after treatment and ask you about a possibility that you experience(d) any pain in the treated area”. In case subjects presented additional question(s), the investigator supplied oral clarifications that had been previously formulated.

The IT-QualST protocol was used as described by Zaguri et al. [12]. A cotton swab (5 mm in diameter) sprayed with Gebauer ethyl chloride was applied immediately to the alveolar mucosa apical to the first premolar on the side of the treated tooth and to the apical mucosa of the first premolar on the contralateral side (3 s on each side, each side tested separately). The swab was located as described by Svensson et al. [14]. The decision to test two sites was made in order to control for a possible bias caused by subjects’ increased awareness of the side that was about to be treated.

Each site underwent two cold stimulations, on two different occasions, as described below. The sequence of the swab application was the treated side/contralateral side on the first encounter and vice versa on the second encounter (contralateral side/treated side).

One investigator (DA) performed all the tests. Prior to the experiment, the procedure of swab spraying with ethyl chloride, swab application, and measurement of the cold sensation duration (see below) was practiced on 11 subjects. As the same examiner performed all the tests, the purpose was to practice and standardize the examiner’s skills in order to achieve maximal intra-examiner reliability. These subjects were not included in the final study.

Subjects were requested to rate the intensity of sensation felt (painful, cold) and the duration of the cold sensation as follows.

The pain and cold sensations were rated using a 10 cm visual analogue scale (VAS). The minimum and maximum possible sensations were defined as “no sensation” and “freezing cold” for cold, and “no pain” and “worst possible pain” for pain. The duration of the cold sensation after the removal of the stimulus (in seconds) was recorded using a stopwatch.

Each patient underwent four IT-QualSTs as follows:

1. On the treated side, immediately prior to the planned restorative treatment. Patients rated the sensation of pain (*Pain-treated1*), sensation of cold (*Cold-treated1*), and duration (in seconds) of the cold sensation (*Time-treated1*).
2. On the contralateral (CL) side, immediately prior to treatment. Patients rated the sensation of pain (*Pain-CL1*), sensation of cold (*Cold-CL1*), and duration (in seconds) of the cold sensation (*Time-CL1*).
3. On the treated side at a follow-up meeting, 1–3 weeks later. Patients rated the sensation of pain (*Pain-treated2*), sensation of cold (*Cold-treated2*), and duration (in seconds) of the cold sensation (*Time-treated2*).
4. On the contralateral side at a follow-up meeting. Patients rated the sensation of pain (*Pain-CL2*), sensation of cold (*Cold-CL2*), and duration (in seconds) of the cold sensation (*Time-CL2*).

#### 2.2.3. Postoperative Dental Pain (PDP), Evaluated through Numeric Rating Scales (NRSs)

Patients were requested to evaluate their PDP at three time points following the restoration: (i) 24 h; (ii) 48 h; and (iii) 72 h.

At each of above time points, patients were contacted via phone and requested to respond to the following questions:

1. What is the level of pain you feel at the treated side at the moment? (NRS ranging from 0—no pain whatsoever to 10—unbearable pain). Results were marked as: *24currentPDP*, *48currentPDP*, and *72currentPDP.*
2. What was the level of the worst pain you experienced in the treated area in the last 24 h? (NRS as above). Results were marked as: *24worstPDP*, *48worstPDP*, and *72worstPDP.*

**Figure 1:**
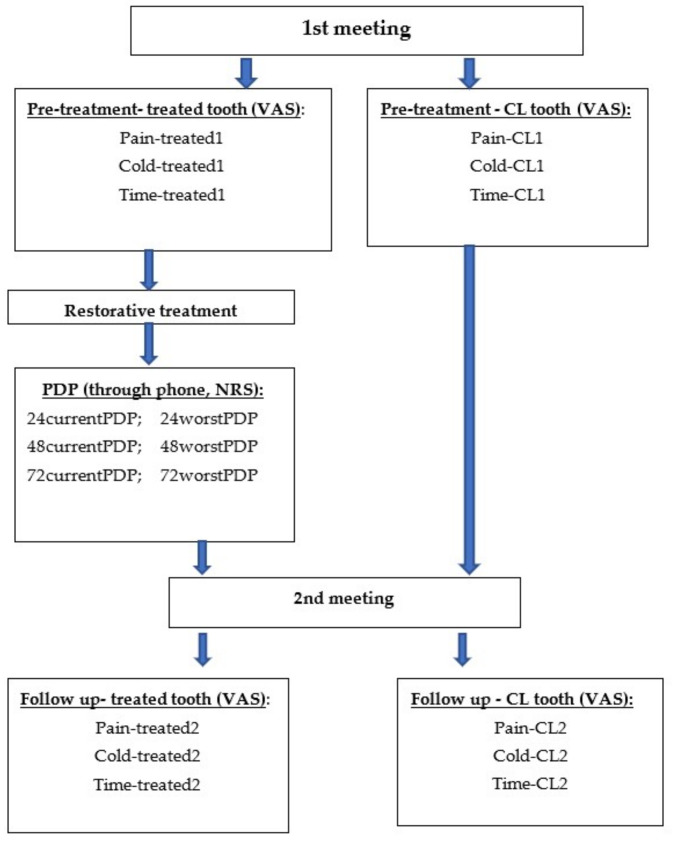
Flowchart of the study protocol.

A flowchart of the study protocol is presented in Figure 1.

### 2.3. Statistical Analyses

The reliability of the IT-QualST measures was assessed through Cronbach’s alpha and Pearson correlation coefficients.

ANOVA with repeated measures (general linear model) was used to compare among PDP at different time points (24, 48, and 72 h).

Stepwise linear regression was used to identify possible predictors of the highest level of PDP experienced by patients.

## 3. Results

### 3.1. Descriptive Data

Power analysis showed that 14 subjects enabled a power of 0.9 in order to achieve a Pearson correlation of 0.75, at a significance level of 0.05, between two normally distributed variables. A bigger group (*n* = 55) was approached in order to allow for the loss of some of the subjects.

Of the initial 55 recruited patients, 13 were excluded due to various reasons (see above). The final study population comprised 42 patients (52% female, mean age 35.6 ± 14.9 years). Subjects’ level of dental anxiety (DAS) was 8.82 ± 4.03.

In total, 59.5% of the performed restorations were in the mandible and 53.7% were Class V. Details about the subjects’ age, type of restoration, and jaw in which the restoration was performed (upper or lower), according to gender, are presented in Table 1. No significant differences were found between any of the above-mentioned variables.

### 3.2. IT-QualST-Pain, Cold, and Cold Duration Assessments

The mean values (±SDs) of subjects’ pain, cold, and cold duration assessments, in the different oral areas (treated side vs. CL side) and at different time points (immediately prior to restoration—first meeting—and at follow-up—second meeting), are presented in Table 2.

No differences could be observed between the treated and CL sides, nor between the first and second meetings. Additionally, no differences were found between the cold assessments (Cold-treated1, Cold-CL1, Cold-treated2, Cold-CL2) nor between the cold duration evaluations (Time-treated1, Time-CL1, Time-treated2, Time-CL2). No differences were found between Pain-treated1 and Pain-CL1 (with the exception of Pain-treated2 versus Pain-CL2, *p* < 0.05).

The reliability of the pain, cold, and cold duration measures was as follows:

(i) Pain (Pain-treated1, Pain-CL1, Pain-treated2, Pain-CL2): Cronbach alpha = 0.84.
(ii) Cold (Cold-treated1, Cold-CL1, Cold-treated2, Cold-CL2): Cronbach alpha = 0.93.
(iii) Cold duration (Time-treated1, Time-CL1, Time-treated2, Time-CL2): Pearson correlation coefficients ranging 0.75–0.80.

As the reliability of the three IT-QualST measures was high, it was decided to use only the measures of the treated side prior to treatment (Pain-treated1, Cold-treated1, and Time-treated1) for further analyses.

### 3.3. Postoperative Dental Pain (PDP)

**Figure 2:**
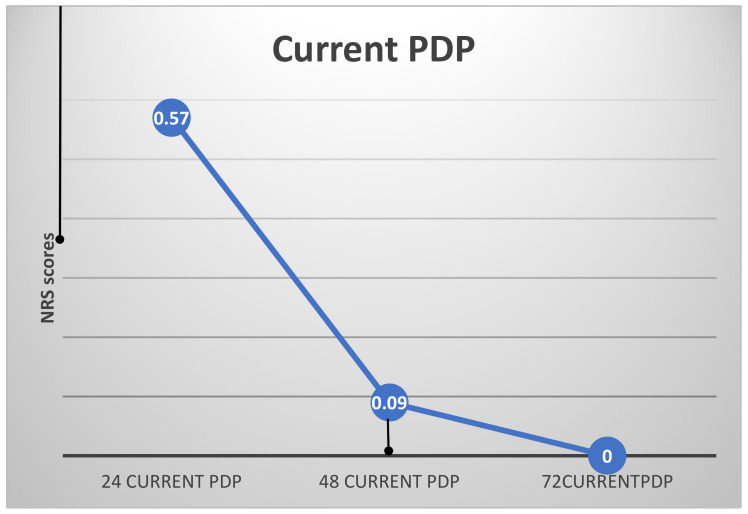
Current PDP (entire population) (mean NRS scores; vertical lines indicate standard error).

**Figure 3:**
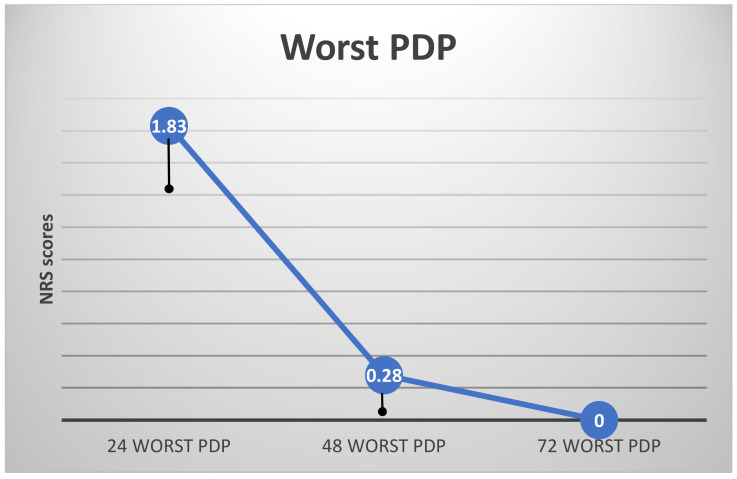
Worst PDP (entire population) (mean NRS scores, vertical lines indicate standard error).

As expected, the highest levels of current and worst PDP were recorded 24 h postoperatively (24currentPDP, 24worstPDP; Figure 2 and Figure 3). There were no differences in subjects’ 24 worst or current PDP in terms of age, gender, type of restoration, or jaw treated (*t*-test).

ANOVA with repeated measures showed a significant effect of time for both current and worst PDP (F_(2)_ = 10.26 and 31.93, respectively, *p* < 0.000 for both) (Figure 2 and Figure 3). The highest PDP was reported 24 h postoperatively (24worstPDP = 1.83 ± 1.92). Both current and worst PDP reached 0 after 72 h. As noted in the Materials and Methods section, patients who reported the use of analgesic drugs postoperatively were excluded from the calculations. Thus, the results indicate subjects’ subjective report with no pharmaceutical influence on their pain experience.

**Figure 4:**
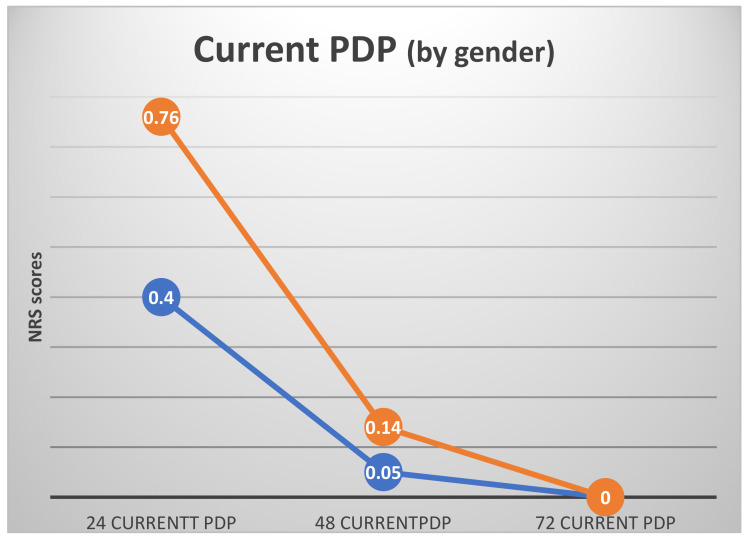
Current PDP by gender with dental anxiety (DAS) as a covariate (mean NRS scores with DAS as a covariate). Covariate appearing in the model evaluated at DAS = 8.8. Females—orange line, males—blue line.

**Figure 5:**
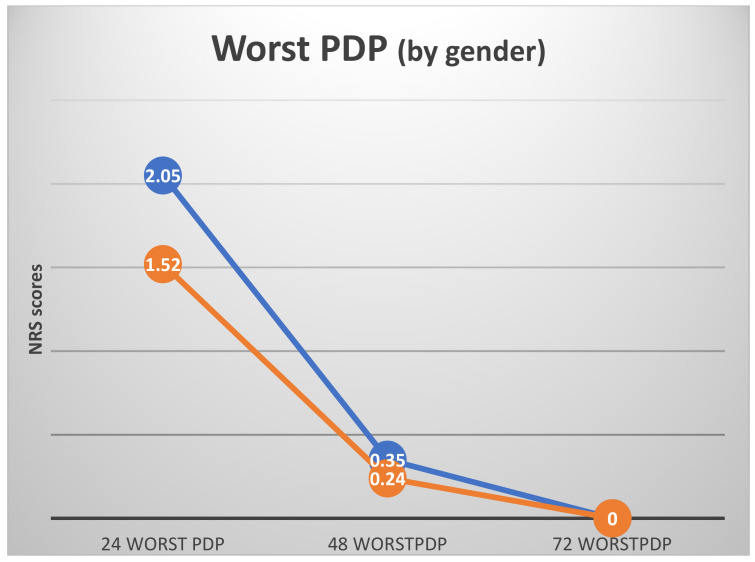
Worst PDP by gender, with dental anxiety (DAS) as a covariate (mean NRS scores with DAS as a covariate). Covariate appearing in the model evaluated at DAS = 8.8. Females—orange line, males—blue line.

When the ANOVA calculations were adjusted for dental anxiety and gender (general linear model by gender, with DAS as a covariate), some differences between genders could be observed. While the general pattern remained unchanged (highest PDP 24 h postoperatively, no PDP 72 h postoperatively), some differences between genders could be observed (Figure 4 and Figure 5).

### 3.4. Stepwise Linear Regressions Analyses

The PDP scores reported by the patients in the present study were rather low (ranging from 0 to 1.83). Therefore, it was decided to examine the factors that could predict the highest level of PDP experienced by patients, namely, their worst PDP 24 h postoperatively. A stepwise linear regression was used to identify possible predictors of 24worstPDP.

Initially, the following candidate variables were entered into the equation: type of restoration (Class I or Class V), jaw treated (maxilla or mandible), Pain-treated1, Cold-treated1, Time-treated1, age, gender, and DAS.

As gender was shown to have a significant effect on pain in the dental setting [32], an interaction between DAS and gender was added to the equation. Due to a possible effect of anxiety on subjects’ perception of pain [32], interactions between each of the three IT-QualST measures and DAS (Pain-treated1*DAS, Cold-treated1*DAS, Time-treated1*DAS) were also included in the analysis. Variables, which emerged as possible predictors of 24worstPDP, were Pain-treated1 and an interaction between male gender and DAS (Table 3a).

On a second step, in an effort to increase the predictive ability of the model, interactions between DAS and each one of the three IT-OualST measures were omitted from the calculation.

Variables entered into the equation were: type of restoration (Class I or Class V), jaw treated (maxilla or mandible), Pain-treated1, Cold-treated1, Time-treated1, age, gender, DAS, and an interaction between DAS and gender (DAS*gender). Under such conditions, variables that emerged as possible predictors of 24worstPDP were Pain-treated1, Time-treated1, and the DAS*gender interaction (Table 3b).

## 4. Discussion

In clinical practice, QST and QualST are used to apply quantitative and qualitative stimuli (temperature, mechanical, electrical, and chemical) to a variety of tissues (e.g., skin, muscle, and viscera), and to use psychophysical methods such as threshold determination or establishing stimulus–response function to assess the function and integrity of the somatosensory system [9].

Cruz-Almedia et al. [13] suggested that with some developments, QST could become a cost-effective and clinically useful component of pain assessment and diagnosis, which can further our progress toward the goal of mechanism-based personalized pain management [13]. Bucci and Michelotti suggested that the application of chairside QualST methods that can be approached with common tools available in a dental clinic should be implemented in daily practice to preliminary detect intraoral sensory disturbances [33]. IT-QualST, with the use of a cotton swab sprayed with ethyl chloride applied to the alveolar mucosa, is a reliable, feasible, cost-effective, and accessible (suitable for everyday clinical use) test that can be used in dental care. The present results suggest that it can be implemented to identify patients prone to developing PDP.

The use of cold stimuli to test pain sensitivity is not a new concept in pain medicine [9,13,34,35]. Its use intra-orally, applied to the dental oral mucosa rather than to a tooth surface in order to test tooth vitality, is a relatively new concept.

Previous studies showed that standardized intraoral QST measures have good inter-examiner and test-retest reliability [36], with no differences between the right and left sides [16]. Similarly, in the present results, no differences were found between the treated and contralateral side. It seems that on the oral mucosa, QST is stable and reliable and can be effective in the assessment of somatosensory function in healthy volunteers, special pain populations, and patients [9].

Most of the studies that have used QST protocols inside the oral cavity have been tested in some chronic dental and oral pain conditions to test for dysfunction in nerve conduction [12,18]. The studies showed its effectiveness in differentiating patients with atypical odontalgia and patients with persistent post endodontic pain [11,17]. In the present study, an attempt was made to check whether such an approach could help clinicians to foretell their patients’ proneness to postoperative pain after restorative dental treatment.

The protocol used in the present study was as described by Zagury et al. [12]. Although using a similar protocol, some differences between the studies emerged. In the present study, the range of pain and cold sensations was relatively low (0.71–1.02 and 3.42–3.71, respectively). This is lower than the scores reported by Zagury et al. [12] in their group of control patients (non-atypical odontalgia). In addition, the duration of the cold sensation in the present study (13.38–15.23 s) was also lower than the one reported by Zagury et al. [12]. Possibly, the differences between studies may be due to age differences [37,38,39].

There are relatively few reviews on age-related changes in the pain sensitivity response. A meta-analysis by Lautenbacher [37] suggested that the pain threshold increases in old age. A more recent systematic review and meta-analysis by El Tumi et al. [39] showed that old adults may be more sensitive to mechanically evoked pain but not thermally evoked pain than young adults. El Tumi et al. [39] recommended the conduction of further studies to compare the pain sensitivity response between different age groups matched for gender, body mass index, ethno-cultural background, socioeconomic variables, and standardized classifications of younger and older age categories. While all of the studies evaluated by El Tumi et al. [39] assessed the pressure pain threshold or heat pain threshold, the stimulus in the present study, and that in Zagury et al. [12], was cold stimulation of the oral gingiva. Moreover, the mean age of the subjects in the present study was 35.6 ± 14.9 years while that of the group of control patients evaluated by Zagury et al. [12] was 45.05 ± 16.55 years, which cannot exactly be classified as young and/or elderly. It is possible that there are differences between the studies other than age (e.g., ethno-cultural background). Further studies are necessary to evaluate this issue.

In addition, the PDP reported by patients in the present study was also rather low (less than 2 on a 0–10 VAS, for the highest PDP experienced). Briso et al. [40] showed that the occurrence of postoperative sensitivity correlates with the complexity of the restoration design and the restorative procedure, both of which can explain the low PDP in the present study in which the restorations were small in size and with minimal penetration into the dentin. The rational for choosing small-sized simple restorative procedures was to reduce operator bias (less sensitivity to the technique) and reduce any possible influence of preoperative pain on the results. Although initially low, the decrease in PDP over time is in accordance with previous publications [1,2,41,42].

Some of the factors that may affect subjects’ experience and report of pain are gender and anxiety. Fear of pain and anxiety sensitivity have consistent influences on pain ratings [40]. Anxious people tend to overestimate the intensity of aversive events such as fear and pain and tend to report more pain after dental treatment [41,42,43,44,45,46].

Previous publications have shown that dental anxiety (DAS) is usually higher among women compared to men [29,47,48]. The present results show that dental anxiety affects PDP report among men in a different way than among women (Gender*DAS interaction). Although men’s DAS scores were lower than those of women’s, their PDP was affected by their dental anxiety while women’s PDP was not.

There is evidence that social models [49] and early learning experiences from social sources such as family and culture [50] may differentially shape gender behavior in men and women. For example, social roles for women may encourage pain awareness and expression, which may increase the willingness of women to report pain and consequently decrease their threshold. In contrast, social roles for men may encourage stoicism and may decrease their pain expression and hence increase their threshold. Defrin et al. [51] showed that the pain threshold (for heat stimulation) of males was not different than that of females but that their pain tolerance was significantly higher than that of females. Furthermore, males presented stronger stereotypic attitudes towards pain sensitivity and willingness to report pain than females. The present results indicate that dental anxiety has a higher impact on men in relation to their PDP report, and that even relatively low levels of dental anxiety can influence men’s postoperative pain report in the dental setting.

The finding that subjects’ sensations, such as the reaction to cold stimulation of the oral mucosa, are significantly associated with the highest PDP experienced 24 h after a restorative procedure is intriguing. The oral mucosa is regarded as a highly developed and specialized sensory system [52]. It can respond to mechanical, thermal, and nociceptive stimuli constituting the somatosensory function [10]. Zagury et al. [12] suggested that in patients with atypical odontalgia, the sensory alterations for cold aftersensation in the oral mucosa indicate a neuropathic mechanism with an involvement of central factors. Subjects in the present study were healthy, non-dentally anxious young adults, experiencing a minimally invasive dental restoration. Regardless, their perception of pain after cold stimulation of their oral mucosa and the duration of the cold sensation after the removal of the stimulus were able to foretell their level of PDP 24 h postoperatively. It is our belief that this method can be used to foretell PDP in other types of restorations and/or dental treatments. Future studies performed on larger groups of patients and including a variety of dental procedures are recommended to further explore this issue.

Effective and safe pain management should be the primary goal of each dental practice. In an era of personalized medicine, it is still an enigma as to which patients will experience PDP. The possibility of using an available, simple, and reliable clinical test for this purpose bears promise. A better understanding of the potential of developing PDP will help clinicians prevent it and better prepare their patients [1].

## 5. Study Limitations

In the present study, all clinical procedures were carried out by dental students under close supervision and with the use of standardized techniques, a fact that might have mitigated some of the possible confounding factors. The minimal size of the restorations could have further minimized the effect of operator variability [53] on PDP. However, while significant, this is a preliminary report, limited to a specific population (young adults) and to minimally invasive restorative dental procedures. This study was carried out on a relatively small group of subjects, a fact that limited the possibility to examine more complex interactions among the studied variables. Further studies are necessary to evaluate the ability of IT-QualST to predict PDP in wider populations and following procedures that are more complex.

## 6. Conclusions

Subjects’ reaction to intra-oral cold stimulation of the oral mucosa can serve as a potential tool to foretell postoperative dental pain following restorative dental procedures. The ability to predict patients who are prone to experiencing PDP will enable clinicians to apply personalized medicine, such as a selective prescribing of postoperative analgesic drugs to certain patients and the avoidance of overprescribing of drugs to patients whose proneness to experiencing PDP is low.

## Figures and Tables

**Table 1:** Distribution of the type of performed restorations and treated jaw according to gender.

Variable		Men (No. = 20)	Women (No. = 22)
Age (years *)		35.30 ± 14.54	35.86 ± 15.59
Type of restoration **	Cl I CL V	52.6% 47.4%	40.9% 59.1%
Jaw treated **:	Upper: Lower	40% 60%	40.9% 59.1%

* mean ± SD; ** percent.

**Table 2:** Mean scores of the QualST pain, cold, and cold duration tests.

QST Measures		Mean	Std. Deviation
Cold evaluation-1st meeting (VAS)	Cold-treated1 *	3.71	2.61
	Cold-CL1 *	3.43	2.57
Cold evaluation-2nd meeting (VAS)	Cold-treated2 *	3.57	2.27
	Cold-CL2	3.42	2.51
Pain-1st meeting (VAS)	Pain-treated1 *	0.71	1.61
	Pain-CL1 *	0.83	1.96
Pain-2nd meeting (VAS)	Pain-trated2 *	1.02	2.11
	Pain-CL2 *	0.76	1.75
Cold duration-1st meeting (seconds)	Time-treated1 **	13.38	13.36
	Time-CL1 **	14.17	15.58
Cold duration-2nd meeting (seconds)	Time-treated2 **	15.23	12.64
	Time CL2 **	14.11	12.97

* VAS (centimeters); ** NRS (seconds).

**Table 3:** Stepwise regression analyses to determine possible predictors of the highest level of PDP experienced by patients (24worstPDP).

(a) Regression model including the following variables: type of restoration, jaw treated, Pain-treated1, Cold-treated1, Time-treated1, age, gender, DAS and the interactions DAS*gender, DAS*Pain-treated1, DAS*Cold-treated1, and DAS*Time-treated1								
					**t**	***p***	**95.0% Confidence Interval for B**	
**Variable**		**B**	**Std. Error**	**Beta**			**Lower** **Bound**	**Upper** **Bound**
Pain-treated1		0.578	0.167	0.491	3.452	**0.001**	0.239	0.917
DAS*gender (male)		0.166	0.065	0.365	2.571	**0.014**	0.035	0.297
(b) Regression model including the following variables: type of restoration, jaw treated, Pain-treated1, Cold-treated1, Time-treated1, age, gender, DAS, and the DAS*gender interaction (final model marked in bold)								
**Model**					**t**	**Sig.**	**95.0% Confidence Interval for B**	
		**B**	**Std. Error**	**Beta**			**Lower** **Bound**	**Upper** **Bound**
1	(Constant)	1.391	0.311		4.470	0.000	0.761	2.021
	Pain-treated1	0.479	0.174	0.408	2.752	0.009	0.127	0.831
2	(Constant)	0.775	0.389		1.991	0.054	−0.014	1.564
	Pain-treated1	0.579	0.169	0.493	3.424	0.002	0.236	0.922
	DAS*gender1	0.160	0.067	0.347	2.406	0.021	0.025	0.295
3	(Constant)	0.236	0.453		0.522	0.605	−0.683	1.156
	**Pain-treated1**	**0.645**	**0.165**	**0.549**	**3.910**	**0.000**	**0.311**	**0.980**
	DAS*gender (male)	**0.136**	**0.065**	**0.293**	**2.092**	**0.044**	**0.004**	**0.267**
	**Time-treated1**	**0.042**	**0.020**	**0.292**	**2.088**	**0.044**	**0.001**	**0.082**

## Data Availability

The data that support the findings of this study are available on request from the corresponding author and are not publicly available due to privacy or ethical restrictions.
